# Traumatic Pericarditis in a Cow; Recovery

**Published:** 1897-07

**Authors:** W. L. Williams

**Affiliations:** Professor of Surgery and Obstetrics, New York State Veterinary College, Ithaca, N. Y.


### REPORTS OF CASES.

### TRAUMATIC PERICARDITIS IN A COW; RECOVERY.

By W. L. Williams, V.S.,
PROFESSOR OF SURGERY AND OBSTETRICS, NEW YORK STATE VETERINARY COLLEGE, ITHACA, N. Y.

There is usually so little to be hoped for in traumatic pericarditis in the cow that the following observation seems worthy of record:

The patient was a valuable Jersey cow in vigorous health and in milk.

About January, 1896, I was called to attend her for tympany, which was moderate and accompanied by painful respirations with grunting during expiration, and a general stiffness and disinclination to move, which led us to suspect the ingestion of a pointed foreign body. The rumen was punctured, saline purgatives with carminatives admiuistered, followed by apparent recovery.

About four weeks later I was recalled, and found the patient dull, stiff, disinclined to move, feverish, dry muzzle, inappetence, tumultuous pulse, intermittent and irregular, with abnormal pericardial sounds. The symptoms were referred to the prior attack of tympany, and traumatic pericarditis diagnosed and an unfavorable prognosis given. Small doses of digitalis were administered three times daily, and within a few days distinct improvement was noted, the appetite returned, febrile symptoms abated, the movements became more free, and the milk flow became normal.

Early in June following I was again requested to examine the animal, and found a prominent tumor beneath and to the right of the sternum in the anterior angle of the space formed by the ensiform and last costal cartilages. The tumor was fluctuating, and, being punctured, about one pint of grayish, very fœtid pus escaped. Exploring the cavity with the finger, two irregular pieces of bone were found, the larger being one-half by three-quarter inches, and sharp. Both were apparently originally of the same piece, and in texture and general appearance had apparently come from a rib or vertebræ, probably from a porterhouse steak, the bone of which had found its way into the kitchen scraps which were fed habitually to the cow.

It seems quite certain that a bone had in this manner been swallowed by the cow, induced the indigestion first noted, penetrated the rumen, passing forward to the right posterior surface of the pericardium, causing pericarditis, after which it travelled to the right and downward, emerging partly decomposed at the point described.

